# On the Importance of Oxidative Folding in the Evolution of Conotoxins: Cysteine Codon Preservation through Gene Duplication and Adaptation

**DOI:** 10.1371/journal.pone.0078456

**Published:** 2013-11-11

**Authors:** Andrew M. Steiner, Grzegorz Bulaj, Nicolas Puillandre

**Affiliations:** 1 Department of Medicinal Chemistry, College of Pharmacy, University of Utah, Salt Lake City, Utah, United States of America; 2 Departement Systematique & Evolution, Museum National d’Histoire Naturelle, Paris, France; The Scripps Research Institute, United States of America

## Abstract

Conotoxin genes are among the most rapidly evolving genes currently known; however, despite the well-established hypervariability of the intercysteine loops, the cysteines demonstrate significant conservation, with a site-specific codon bias for each cysteine in a family of conotoxins. Herein we present a novel rationale behind the codon-level conservation of the cysteines that comprise the disulfide scaffold. We analyze cysteine codon conservation using an internal reference and phylogenetic tools; our results suggest that the established codon conservation can be explained as the result of selective pressures linked to the production efficiency and folding of conotoxins, driving the conservation of cysteine at the amino-acid level. The preservation of cysteine has resulted in maintenance of the ancestral codon in most of the daughter lineages, despite the hypervariability of adjacent residues. We propose that the selective pressures acting on the venom components of cone snails involve an interplay of biosynthetic efficiency, activity at the target receptor and the importance of that activity to effective prey immobilization. Functional redundancy in the venom can thus serve as a buffer for the energy expenditure of venom production.

## Introduction

Conotoxins are peptides from the venom of *Conus*, a genus of predatory marine gastropod. When a cone snail envenomates its prey, a very small volume (6–480 *μ*l; avg. 56.21 *μ*l for *C. purpurascens*) [1] of a highly-diverse venom is injected to paralyze the prey. This highly-diverse venom is largely composed of disulfide-rich peptides, which primarily target ion channels on the cell surface [2]. The extraordinary diversity of *Conus* venom is genetically-encoded within the hypervariable mature toxin region [3], and arises from gene duplication and positive selection [4], which are believed to be at constant work in *Conus*, allowing the snail to compete effectively in the venom-target molecular arms race [5]. This results in exceptionally potent and highly specific toxins, such that only a small volume of venom is necessary to efficiently paralyze the prey. The high potency of conotoxins minimizes the venom requirements (thus, energetic expenditure) of each envenomation event, while also allowing some variation in targeted prey.

Conotoxins have been extensively studied with respect to function [6], oxidative folding [7], gene structure [3], [4], [11], and their evolutionary patterns [8]–[10]. Conotoxin genes consist of a signal sequence, propeptide, and a toxin region containing highly conserved cysteine residues separated by hypervariable intercysteine loops. In this work we focused on the 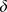
- and 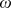
- families of conotoxins, which bear the intercysteine knot (ICK) motif. The ICK motif is defined by the cysteine scaffold, which consists of six total cysteines with a single pair of central vicinal cysteines (C–C–CC–C–C). Both 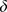
- and 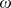-conotoxins are short and cysteine rich; the mature peptide is generally 25–35 residues long. The ICK motif is also broadly employed by many organisms outside *Conus*, including insects, plants, fungi and spiders [12]; previous work has also referred to conotoxins bearing the ICK motif as ‘four-loop conotoxins’ [11], [13], [14].

It is widely believed that the hypervariability of the intercysteine loops results from positive selection after gene duplication [15]–[17], based on the activity of the conotoxin at the target receptor [4], [5], [18], [19]; it follows that a mutation which confers a selective advantage with respect to activity will be preferentially retained over a mutation which is either neutral or detrimental to the activity. While activity does play a major role, the production/folding efficiency is also likely to be a critical factor; both could direct the evolution of the amino acid sequences of the intercysteine loops, as well as the preservation of disulfide scaffolds and post-translational modifications. Occasionally, the latter two are significant only to folding efficiency [20]–[22], although many changes affect both production efficiency and activity. Consequently, we propose that both activity and production efficiency be considered in models of conotoxin evolution.

Highly conserved cysteine residues are interspersed with the hypervariable intercysteine loops in conotoxins, and there is a site-specific codon preference for each cysteine in the disulfide scaffold [13], [23]. However, the driving force behind the extensive site-specific conservation of cysteine codons–which are flanked by hypervariable intercysteine loops–has not yet been fully elucidated. Previous reports suggested that this site-specific cysteine codon bias could result from a ‘block substitution’ mechanism [23], or a macromolecule that binds specifically to TGC or TGT [13], [24]; the latter has received support from frame-shifted pseudogenes which retained characteristic cysteine codons [13], [25]. However, we hypothesize that the codon level conservation is driven by conservation of cysteine at the amino-acid level, and speaks more to the evolutionary history of these genes than any explicit mechanism driving conservation at the codon level.

To that end, and considering results that demonstrated that *in vivo* conotoxin folding is not quantitiatve [26], we first analyzed the extent to which chaperones can expend energy to modulate the outcome of oxidative folding. Microsome-assisted folding is an established method to consider the role that chaperones play in folding; microsomes have been used to quantify chaperones in the endoplasmic reticulum [27], analyze the dynamics of the interactions between chaperones and the unfolded protein load [28], and assess the role of chaperones in efficient oxidative folding [26]. The microsomes offer a representative sample of the chaperones that are in the endoplasmic reticulum, and can be added in varying concentrations as a component of a folding reaction. By comparing the folding kinetics in the presence of untreated microsomes versus ATP-depleted microsomes, we assessed the extent to which chaperones can expend energy to modulate the outcome of oxidative folding, and found that the intrinsic propensity for the native disulfide connectivity is the dominant factor in biosynthetic efficiency. We then collected statistics on all conotoxin sequences that could be reliably classified into the 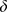 and 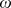 families. These sequences were analyzed for their cysteine codon bias, the extent of conservation of out-of-frame cysteine residues, and were also used with a novel analysis tool that enables comparison of codon bias independent of the encoded residue, allowing meaningful comparison between cysteine sites and the hypervariable intercysteine loops. By using an internal reference, we are effectively correcting for the homology of these gene families. No correction for homology was present in previous statistical analyses of cysteine codon bias in conotoxins [13]. The picture that emerges is one of amino-acid level conservation of cysteine, which is further supported by phylogenetic analyses, showing that once a silent mutation occurs at a cysteine residue, there is no reversion bias to the canonical cysteine codon.

In light of the critical role that innate folding efficiency plays in biosynthetic efficiency, we propose that folding efficiency is one of the critical factors directing the evolution of conotoxins, and that cysteine codons are maintained due to the importance of each cysteine in an existing disulfide scaffold. This would imply that the only selective pressure–combined with evolutionary diversification from one (or a few) ancestral gene(s)–is at the amino acid level, and that the observed codon conservation [13], [23]–[25] is an artifact of the combination of cysteine conservation through selective pressure and phylogenetic conservatism.

## Results and Discussion

### Chaperone-assisted Oxidative Folding

There is an established role for energy expenditure in efficient oxidative folding of proteins [29]–[31], which likely functions through BiP, the ER-resident 70 kDa heat shock protein [32]. However, the extent to which this applies to small substrates–such as conotoxins–is unclear. The recent discovery of misfolded ImI in *Conus imperialis* venom [26] established that *in vivo* folding faces many of the same challenges to production efficiency as *in vitro* folding, indicating that the ability of chaperones to expend energy to modulate the outcome of oxidative folding remained an open question.

To assess the extent to which chaperones expend energy to direct the oxidative folding of conotoxins, we considered the ATP-dependence of chaperone-assisted folding using microsomes derived from rat liver (see Materials & Methods). These results are shown in Table 1, and demonstrate a minor ATP-dependence for the folding kinetics of 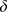-PVIA and 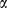-ImI, although the oxidative folding of 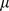-SIIIA appears to be ATP-independent. HPLC traces which show the differences between ATP-depleted microsomes and untreated microsomes are shown in File S1 (Figures S3, S4 and S5). For the two conotoxins that did show ATP-based acceleration of folding kinetics, the effect was fairly subtle, suggesting that the peptide’s inherent propensity for the native disulfide connectivity is likely to be the dominant factor determining the *in vivo* folding efficiency.

**Table 1:** ATP-dependence of microsome-assisted folding.

Microsome-Assisted Folding				
		Amount Native (%)		
Peptide	Folding Time	ATP-Depleted Micros	Untreated Micros	No Micros
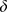 -PVIA*	24 hours	4.63±0.09%	5.07±0.33%	0%†
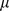 -SIIIA	30 min	2.09±0.77%	2.89±0.21%	1.02±0.31%
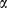 -ImI*	8 min	1.62±0.18%	1.93±0.046%	0.420±0.017%

As a model of *in vivo* oxidative folding in the ER, microsomes derived from rat liver were used to fold linear peptide. Only the timepoint with the largest difference is shown. Values given are Mean ± Standard Deviation (n = 3).

† No native peptide could be detected in the indicated condition.

* Indicates that this peptide showed a statistically significant difference between microsomes that were ATP-depleted and untreated microsomes (paired, one-tailed t-test of three independent experiments; P < 0.05).

The inherent propensity for the native disulfide connectivity also has several layers, including the disulfide bonds themselves and post-translational modifications. In addition to holding the peptide in an active conformation [33]–[35], disulfides can also be critical to folding efficiency. In 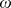-GVIA, removing the Cys1–Cys16 disulfide bridge negligibly affects its activity on N-type calcium channels, but greatly reduces the peptide’s propensity for the native connectivity, despite reducing the number of possible disulfide isomers from fifteen to three [20]. Consequently, a disulfide can be conserved in order to preserve activity, or to preserve efficient folding, or both. It remains unclear whether a disulfide that is valuable only to folding efficiency acts by conformationally constraining the peptide as the other bridges form, or whether that cysteine pair drives thiol/disulfide exchange through a more productive folding pathway.

Post-translational modifications can also function to increase folding efficiency, without any measurable change in activity of the peptide. For instance, hydroxylation of proline in 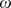-MVIIC provides a 2-fold increase in folding rate, as well as a 3-fold increase in yield, but does not change the activity (as measured by intracerebroventricular injection into mice) [21]. Also, the 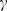-carboxylation of glutamate can introduce calcium-dependent improvements to folding efficiency, without measurable effects on activity (also measured by intracerebroventricular injection into mice) [22]. While chaperones can offer some benefit to folding efficiency *in vivo*, their enhancement is largely limited to kinetics, as enzymes lower energy barriers, but do not change equilibria, although it is possible that kinetic enhancement to productive folding pathways could be coupled to secretion from the endoplasmic reticulum to effect a non-equilibrium folding outcome.

### Cysteine Codon Bias in Conotoxins

**Figure 1:**
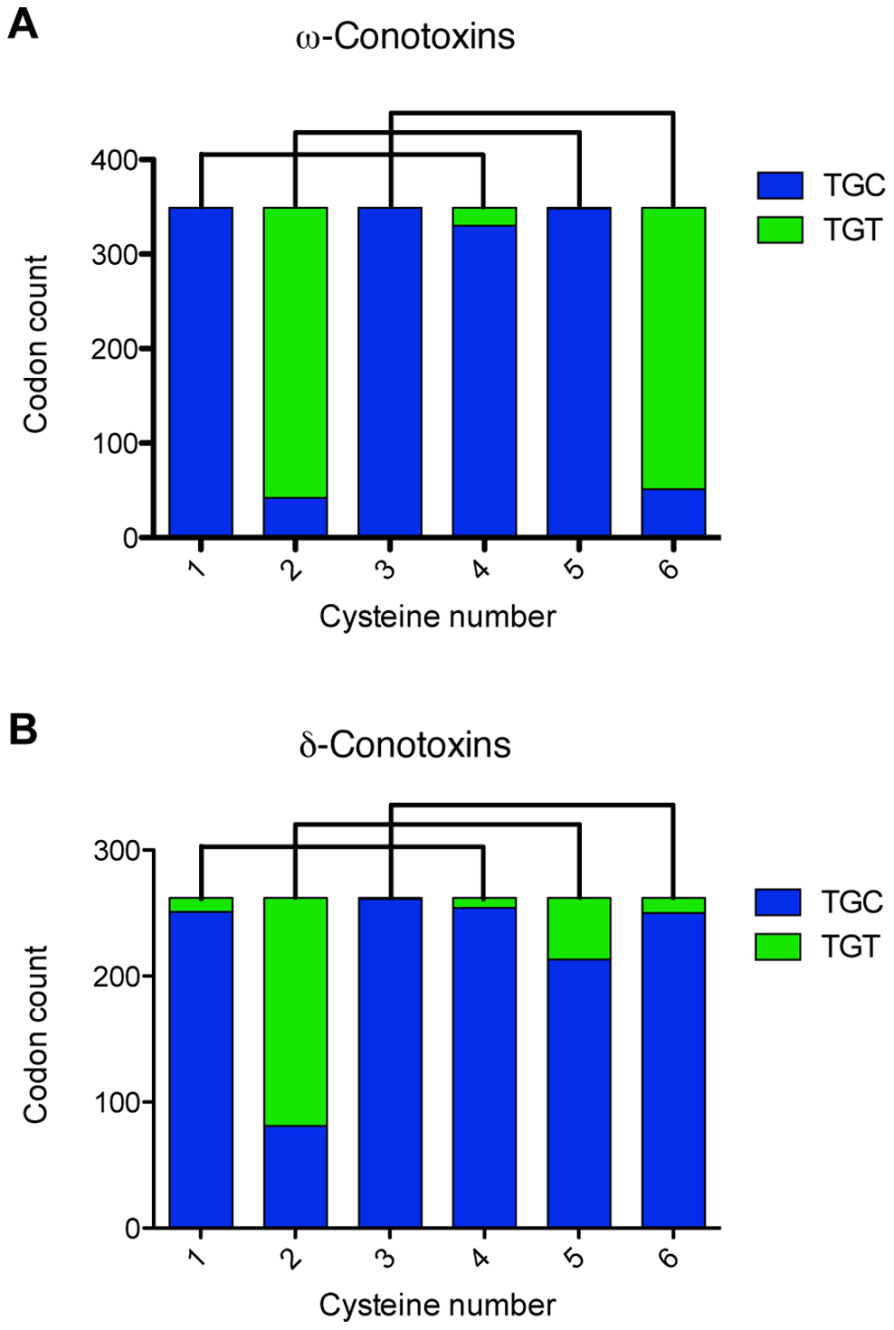
Codon usage statistics for the cysteine residues in two classes of four-loop conotoxin genes. (**A**) shows the codon bias for the 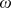-conotoxins, and (**B**) shows the codon bias for the 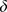-conotoxins. The lines above show the characteristic disulfide connectivity of the cysteine residues. While both 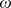
- and 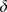-conotoxins maintain the same general cysteine scaffold, they are readily distinguished by the precursor sequences (both signal and propeptide), length of the intercysteine loops, as well as the tendency of the 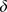-conotoxins to be considerably more hydrophobic. The GenBank accession numbers of all sequences used are available in File S1.

Published 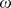
- and 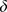
- conotoxin sequences were analyzed for the extent of cysteine codon bias. To ensure that each sequence was properly classified in the 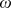 or 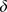 family, only sequences containing the signal sequence, propeptide and mature toxin region were analyzed; duplicates were also removed. It is important to note that the classification as a 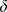
- or 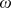-conotoxin is made based on the pharmacological activity of the peptide (acting to either delay inactivation of sodium channels or block the pore of calcium channels, respectively [36], [37]), and not on the basis of genetics. Both 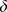
- and 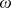-conotoxins are members of the O1 Superfamily of conotoxin genes. Since the vast majority of the sequences employed in this study have never been synthesized and pharmacologically analyzed, the classification into the 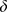 or 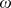 peptide family is based on characteristic patterns in the signal sequence, established by comparison to peptides of known pharmacology. The observed codon bias for cysteine sites in the resultant 349 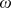
- and 262 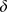
- conotoxin sequences is shown in Figure 1, which also shows the characteristic disulfide connectivity of both families. GenBank accession numbers of all sequences used in this analysis are shown in File S1.

In concurrence with previous results, we saw remarkable site-specific codon bias for cysteine, which differed between the 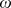-conotoxins and the 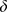-conotoxins at the final cysteine residue (Figure 1). However, the extent to which the observed codon conservation is actually functioning at the codon level remains unclear. It is entirely plausible that the codon conservation speaks more to the evolutionary history of the O1-Superfamily of conotoxin genes–and the significant pressure to preserve cysteine due to its role in oxidative folding–than to any explicit mechanism of cysteine codon conservation.

### Out of Frame Cysteine Codons

**Figure 2:**
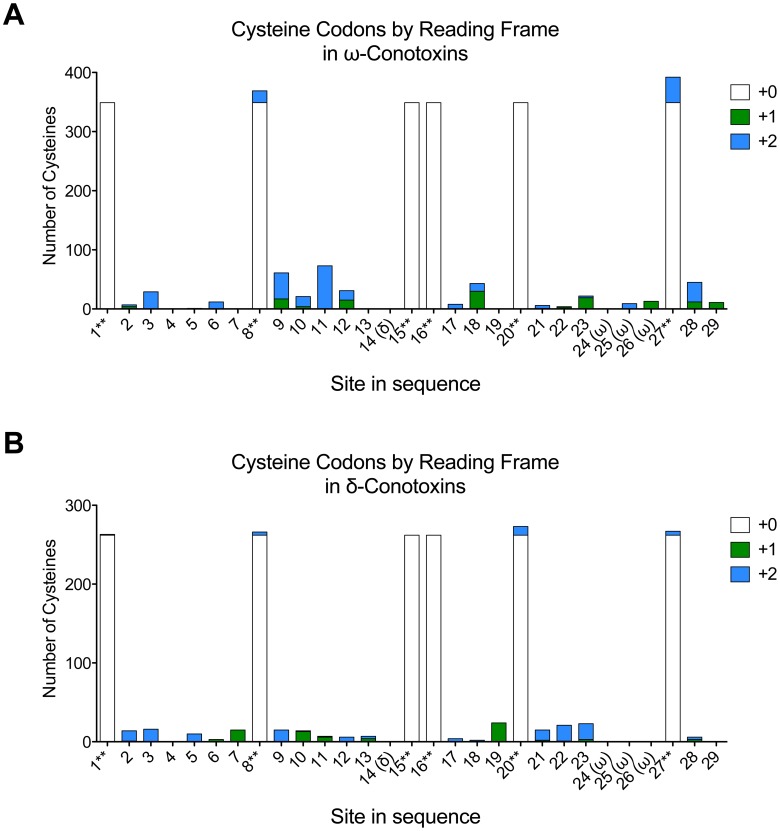
Comparison of in-frame and out-of-frame cysteine codons for 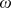
- and 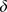-conotoxins. (**A**) shows the data for the 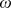-conotoxins, and (**B**) shows the data for the 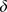-conotoxins. White bars show in-frame cysteine codons, green bars show cysteine codons in the +1 position, and blue bars show cysteine codons in the +2 position. ‘Site in sequence’ starts counting at the first cysteine in the mature toxin region, and considers only sites that have at least 100 sequences in the alignment. These numeric designations are preserved in Figures 3, S1 and S2. Sites that are specific to one class of sequence are marked with that class (e.g. 14(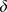) contains data for only the 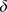-conotoxins). Cysteine sites are marked with **; these sites are also readily identifiable by the large white bars that indicate in-frame cysteine codons.

As previous reports have used the conservation of out-of-frame cysteine codons to validate a direct mechanism for codon-level conservation of cysteine in conotoxins, we also analyzed the out-of-frame cysteine codons, using the same dataset of 349 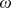-conotoxins and 262 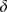-conotoxins. The results for each family are shown separately in Figure 2.

While Figure 2 shows that there is generally very little conservation of out-of-frame cysteine codons, there does appear to be minor conservation of out of frame cysteine codons at sites 9, 11, 18 and 28 in the 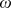-conotoxins; however, this constitutes only a small fraction of the total number of sequences. Consequently, we believe that out-of-frame cysteine codons speak more to the evolutionary history of the O1-Superfamily of conotoxin genes than to any direct mechanism driving conservation of cysteine codons.

### Codon Bias Analyses

Because oxidative folding is a critical step in conotoxin biosynthesis, the high degree of cysteine conservation is likely indicative of its significance to biosynthetic efficiency; when a disulfide bridge that is dispensible with respect to activity is removed from 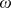-GVIA, the folding yield decreases more than two fold [20]. Accordingly, the loss of a cysteine residue due to a mutation or frameshift could be significantly more detrimental than mutations that result in changes to the residues in the intercysteine loops. For instance, if one cysteine is removed, then the peptide will either create a disulfide dimer (homo- or hetero-dimer) [38], or it will retain a free thiol, leading to inter- and intra-molecular disulfide shuffling in the venom duct, after secretion away from ER-based quality control machinery. If a pair of cysteines is lost, the resulting disulfide scaffold may loose activity, or may have a dramatically decreased folding efficiency [20]. Conversely, if a mutation changes a non-cysteine residue, the probability of retaining the important chemical attribute(s) is much greater. For example, if a given site must have a negatively charged amino acid for folding/activity, then there are two possibilities (D and E), encoded by four codons (GAT, GAC, GAA and GAG). Considering only single-base substitutions, then the probability to retain an amino acid that is negatively charged is one in three, or 33.3%. Since cysteine is the only residue capable of forming crosslinks, and can be encoded by two codons (TGT and TGC), the probability of retaining this functionality through a single-base substitution event is one in nine, or 11.1%. Hence, if a point mutation occurred, it would be much more likely to be detrimental at a cysteine site than elsewhere in the peptide because only silent mutations conserve the existing disulfide scaffold, effectively selecting against non-silent mutation at cysteine sites.

**Figure 3:**
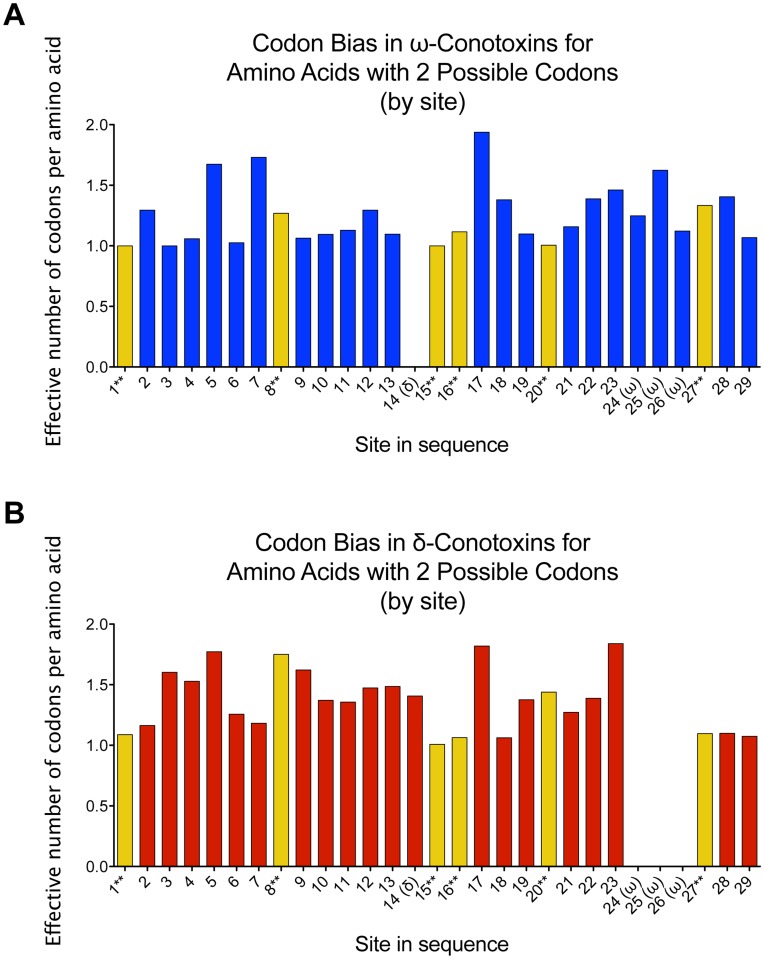
Codon bias for 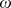
- and 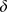-conotoxins. (**A**) shows the data for the 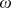-conotoxins, and (**B**) shows the data for the 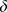-conotoxins. Values presented are the weighted average of 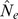 values (*sensu* [43], Eq. 2) for codons encoding amino acids that can be represented by two (and only two) codons. Because the number of residues in the intercysteine loops differ between the 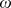
- and 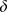-Conotoxins, sites that are specific to one class of conotoxins are followed by the class to which they are specific (e.g. site 14(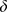) only shows data for 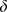-conotoxins). Yellow bars and **indicate cysteine sites. Analysis was only conducted on sites with at least one hundred sequences in the alignment, at least thirty of which were residues that could be encoded by two codons. A value of 1 indicates that only a single codon is used, and a value of 2 indicates that there is no codon bias among the two possible codons for each residue. The same data for the other redundancy classes is presented in File S1 (Figures S1 and S2), along with the GenBank accession number of each sequence included in the analysis.

To reexamine the codon conservation of cysteine in conotoxins, we sought a means to assess codon bias at each site that did not account for the encoded amino acid, in order to meaningfully compare codon bias at cysteine to neighboring hypervariable residues. By using an internal reference for codon bias, we accounted for the homology between sequences, allowing facile distinction between codon-level and amino-acid-level conservation. To accomplish this, we adapted the “effective number of codons” [39] to consider a group of related sequences site-by-site. In order to avoid assumptions regarding amino acids that were missing or underrepresented, we took a weighted average of the codon bias among amino acids that can be represented by the same number of codons (a ‘redundancy class’), allowing analysis of codon bias independent of the amino acid encoded by each codon (details in Materials & Methods). The codon bias for each cysteine is compared to the codon bias in the intercysteine loops for other residues that can be encoded by two codons in Figure 3. Thus, when we accounted for homology among the members of a conotoxin family, the codon bias for cysteine became rather subtle, indicative of amino acid level conservation, rather than site-specific bias for a particular cysteine codon.

### Phylogenetic Analyses

**Figure 4:**
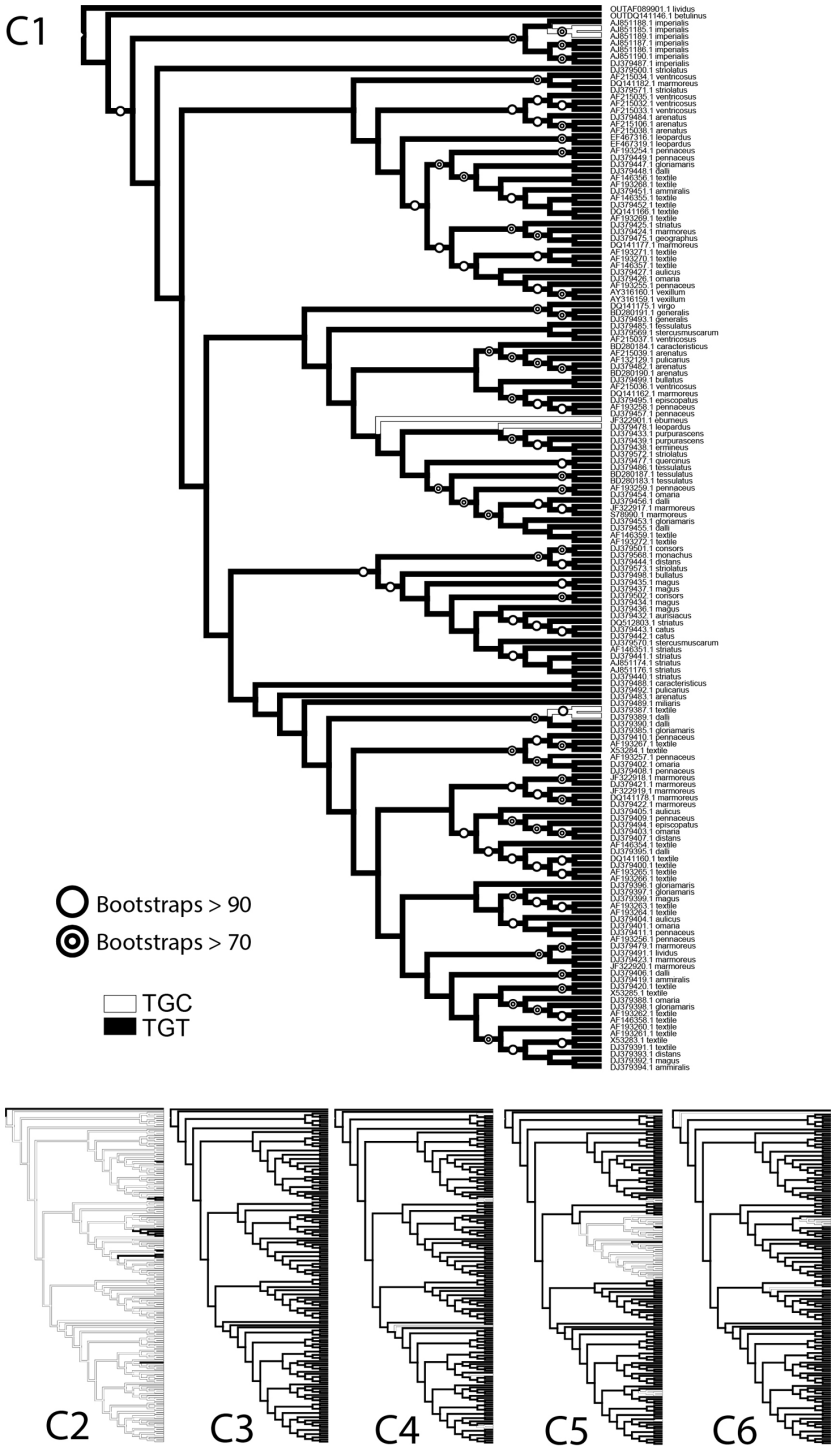
Phylogenetic trees of a represenatitve subclade of 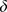-conotoxin genes used in this study, showing the codon usage patterns for each cysteine. Each tree shows the codon usage for a single cysteine, and is labeled accordingly. Shaded lines indicate TGT, and hollow lines indicate TGC. Bootstrap values are also shown on the tree for Cysteine 1. Each branch in the tree for Cysteine 1 is labeled with the GenBank accession number of the sequence and the *Conus* species. Phylogenetic trees for the entire O1-Superfamily are shown in File S1.

An alternate means to account for homology among members of a conotoxin family is by use of phylogenetic analyses. Under the assumption that the O1-Superfamily of conotoxin genes evolved from a single ancestral sequence, we constructed a phylogenetic tree to chart the divergence of these genes. Figure 4 shows the phylogenetic tree for a representative subclade of the 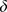-conotoxins; the trees for all sequences used in this study are shown in File S1 (Figures S6–S11). Mapping cysteine codon usage onto the tree results in six copies of the tree, each marking codon usage for a particular cysteine (Figure 4, C1–C6). The trees for cysteines 2 and 5, in particular (but see also File S1), clearly show that mutations from one codon to the other arose independently several times; furthermore, in several cases (e.g. Figure 4 C5 tree, TGC clade, in white), once a mutation occurs, it is transmitted to the descendant sequences, suggesting that there is no counter-selection to revert to the canonical cysteine codon at that site. Taken together, these results are compatible with a scenario where cysteine is conserved at the amino acid level, and where the observed, but weak, codon level conservation is the result of a phylogenetic conservatism linked to the strength of the evolutionary pressure to preserve cysteine. In the M-Superfamily of conotoxins, it has been proposed that the divergence into distinct branches with distinct disulfide connectivities (and length of intercysteine loops) was concurrent with a switch in codon-preference at each site [40], further supporting that the cysteine codons are retained by evolutionary constraints on folding, and that the evolutionary pressure likely acts to retain cysteine residues, but does not act directly on the codon level.

### Significance & Implications

Where it has been previously assumed that activity is the driving factor for the evolution of conotoxins [4], [15], [18], [19], we propose that the evolutionary pressures acting on the venom components of cone snails involve an interplay of production/folding efficiency, activity at the target receptor, and the relative importance of that activity to effective prey immobilization. Mutations that enhance or detriment folding efficiency or activity can be assessed with respect to energetic cost per activity unit, allowing a combined analysis of the potential cost and value of a mutation with respect to the evolutionary pressures that cone snails experience. For example, if a peptide with an indispensable activity is mutated such that it is two-fold more active, but folds one fourth as efficiently, then the snail must produce two times as much of that peptide (costing twice the energy) to effect the same result upon envenomation. However, in reality, the functional redundancy of *Conus* venom (for example, the 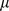-conotoxins BuIIIA, BuIIIB and BuIIIC in *Conus bullatus*, [41]) likely serves to dampen this variability, effectively buffering the energetic cost of venom production against drastic perturbations that result from changes to the folding efficiency or activity of individual conotoxins. Because mutations at cysteine residues are more likely to result in decreased folding efficiency and/or activity, there is likely significant pressure to retain cysteine residues, which in turn results in substantial codon bias over the course of evolutionary diversification of conotoxin gene families associated to phylogenetic conservatism. Using a measure that corrects for homology among conotoxin genes, we have shown that the established codon bias is more likely to speak to the evolutionary history of the conotoxin genes than explicit codon-level conservation of cysteine. Thus, we believe that the site-specific codon-level conservation of cysteine is driven by amino-acid level conservation of cysteine’s unique properties, and the importance of efficient oxidative folding in conotoxin biosynthesis.

Since folding efficiency appears to be a driving factor for the evolution of conotoxin genes, and a protein’s amino acid sequence dictates that protein’s folded structure [42], we postulate that changes to most protein sequences must preserve efficient *in vivo* folding. While the preservation of cysteine allows us to readily test this hypothesis in conotoxins, most proteins lack the numerous disulfide crosslinks present in conotoxins, and are therefore much more difficult to assay for the significance of individual residues to the overall folding efficiency. Additionally, chaperones may play a more significant role in directing protein folding than in the oxidative folding of smaller substrates, such as conotoxins. Nonetheless, as proper folding is crucial for activity [43], the *in vivo* protein folding efficiency of most proteins is likely to be as indispensable as the protein itself.

## Materials and Methods

### Microsome-assisted Folding

Microsomes were prepared as in [26]. Briefly, liver tissue from a male Sprague-Dawley rat was homogenized in a buffer containing 20 mM Tris, pH 7.5, 50 mM NaCl and EDTA-free protease inhibitor cocktail (Roche), using 1 ml homogenization buffer per 100 mg of tissue in a dounce homogenizer. The homogenized material was centrifuged at 12,000×g for 10 minutes. The supernatant was then centrifuged at 100,000×g for 1 hour; the supernatant was removed and the pellet was resuspended in fresh homogenization buffer. This was then centrifuged again at 100,000×g for 1 hour. The supernatant was again removed, and the washing process and centrifugation were repeated. The pellet was then resuspended using sodium carbonate buffer (approximately pH 11), and set on ice for twenty minutes to release the contents of the microsomes. This material was centrifuged at 100,000×g, and the supernatant was then removed, pooled, and the pH was adjusted to neutral with HCl. Total protein was then quantified with Bradford Reagent (Sigma), according to the manufacturer’s instructions, using bovine serum albumin as a standard.

Microsome-assisted folding reactions were conducted with 40 *μ*g microsomal protein per 1.5 nmol of peptide, in a Tris-buffered solution (0.1 M final concentration, pH 7.5) containing 1 *μ*M glutathione disulfide. ATP-depletion was accomplished as in [31]. Briefly, we pre-treated microsomes with potato apyrase (New England BioLabs) in the provided succinate buffer at 30°C for 30 minutes. Control reactions were similarly treated, using apyrase storage buffer without apyrase. Reduced peptide was added to commence the folding reactions (20 *μ*M final concentration), and timepoints (1.5 nmol peptide) were removed at regular intervals and quenched by addition to a tube containing formic acid (8.5% final–resulting in a final pH of approximately 1.78 [44]). Samples were then analyzed by reverse-phase high performance liquid chromatography using a diphenyl column (219TP54, Vydac), and were eluted using a linear gradient of acetonitrile in water, maintaining 0.1% trifluoroacetic acid (TFA) throughout. Chromatograms were analyzed by peak integration; the natively-folded peak was established using a reference standard of folded, active peptide.

### Statistical Analyses

Significance tests on microsome-assisted folding reactions were performed in Prism 5.0 d, using a paired, one-tailed t-test of three independent experiments and a significance threshold of 0.05.

The development of the statistical tool to assess codon bias independent of the encoded amino acid is discussed in detail (including both theoretical development and a sample calculation) in File S1. Briefly, we adapted the ‘effective number of codons’ [39] to assess a single site in a group of homologous genes (rather than every site of a single gene). This was accomplished by grouping the data in the sequence alignment vertically, and using the set of codons employed for that site to determine 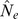 values for each amino acid (as in [39], Eq. 2). The 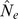 value in this case is the number of equally frequent codons that would produce the observed level of homozygosity, or the effective number of codons for that amino acid. Where Wright consolidated the 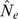 values for each amino acid to a single number [39], this consolidation involved assumptions about amino acids that are missing or underrepresented. As the cysteine sites in our alignment contained only cysteine, we sought to avoid assumptions about amino acids that were missing or underrepresented. So, we elected to take the weighted average of the 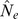 values; while this only allows comparison within a redundancy class (for instance, cysteine can only be compared to other residues that can be encoded by two and only two codons), it does allow meaningful comparison between the cysteine sites and the intercysteine loops because the codon bias is assessed independent of the encoded amino acid. However, it is worth noting that the restriction to the nine residues that can be encoded by two and only two codons (C, N, D, Q, E, H, K, F, Y) does provide a smaller sample size for the intercysteine loops than the cysteine sites; only sites with at least 100 sequences in the alignment, 30 of which contained amino acids that are encoded by two codons, were included in the analysis.

### Phylogenetic Analyses

Phylogenetic tree reconstruction was performed on the Cipres Science Gateway (http://www.phylo.org/portal2), using RAxML-HPC2 on TG tool; robustness of the nodes was assessed with a bootstrap analysis (1000 replicates). The evolution of cysteine codons was assessed with Mesquite V2.74 [45], using the option “tracing character history” and the parisomony ancestral reconstruction method. Each tree (both in Figure 4 and Figures S6–S11 in File S1) shows the codon usage for a single cysteine.

### Ethics Statement

This study was carried out in accordance with the recommendations set forth in the Guide for the Care and Use of Laboratory Animals of the NIH. To minimize suffering, liver tissue from a single rat was used for all experiments. The rat was euthanized and tissue harvested in accordance with a protocol approved by the University of Utah Office of Research Integrity and Compliance (IACUC Protocol# 11-09010).

## Supporting Information

File S1
**This Supporting Information file (PDF) includes Figures S1–S11 and Tables S1–S4; it also contains a thorough discussion of the codon bias analysis methods and lists the sequences used for analysis.** The rationale and methods for the codon bias analysis are presented first (including Tables S1–S4, showing sample calculations), along with the presentation of codon bias for the codons that are 4- or 6-fold redundant (Figures S1 and S2, respectively). Representative HPLC chromatograms from the folding reactions of 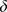-PVIA (Figure S3), 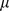-SIIIA (Figure S4) and 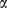-ImI (Figure S5) follow. The complete phylogenetic trees of all sequences used in the analysis are presented in Figures S6–S11, with each figure showing the tree for a single cysteine. The accession numbers of each sequence used in the analysis is also included.(PDF)
