# Supplementary material for: On the Importance of Oxidative Folding in the Evolution of Conotoxins: Cysteine Codon Preservation through Gene Duplication and Adaptation
Source: PLoS One. 2013 Nov 11;8(11):e78456. doi: 10.1371/journal.pone.0078456 (PMC3823881; doi:10.1371/journal.pone.0078456)
Supplement: File S1 — This Supporting Information file (PDF) includes Figures S1–S11 and Tables S1–S4; it also contains a thorough discussion of the codon bias analysis methods and lists the sequences used for analysis. The rationale and methods for the codon bias analysis are presented first (including Tables S1–S4, showing sample calculations), along with the presentation of codon bias for the codons that are 4- or 6-fold redundant (Figures S1 and S2, respectively). Representative HPLC chromatograms from the folding reactions of -PVIA (Figure S3), -SIIIA (Figure S4) and -ImI (Figure S5) follow. The complete phylogenetic trees of all sequences used in the analysis are presented in Figures S6–S11, with each figure showing the tree for a single cysteine. The accession numbers of each sequence used in the analysis is also included. (PDF) [file pone.0078456.s001.pdf]

Supporting Information:
On the Importance of Oxidative Folding in the Evolution of Conotoxins: Cysteine Codon Preservation Through Gene Duplication and Adaptation

Andrew M. Steiner[1,*], Grzegorz Bulaj[1], Nicolas Puillandre[2]

1 Department of Medicinal Chemistry, College of Pharmacy, University of Utah, Salt Lake City, Utah, United States

2 Departement Systematique & Evolution, Museum National d'Histoire Naturelle, Paris, France

∗ **E-mail: steiner.andrew@utah.edu**

Contents

S1 Determination of the Effective Number of Codons per Amino Acid by Site S3

S1.1 Rationale . . . S3

S1.2 Overview of Adaptations to Effective Number of Codons . . . S4

S1.3 Theoretical Buildup . . . S5

S1.4 Sample Calculation . . . S6

S1.5 Data for Redundancy Classes 4 and 6 . . . S8

S2 HPLC Data From Microsome-Assisted Folding S10

S3 Phylogenetic Trees for O1 Superfamily S13

S4 Sequences Used for Analysis S15

S4.1 ω-Conotoxin Sequences . . . S15

S4.2 δ-Conotoxin Sequences . . . S23

Supplemental References S25

List of Figures

S1 Codon bias in the ω- and δ-conotoxins, showing the bias for amino acids that can be represented by 4 possible codons. S11

S2 Codon bias in the ω- and δ-conotoxins, showing the bias for amino acids that can be represented by 6 possible codons. S12

S3 HPLC Chromatograms for folding reactions of δ-PVIA. S13

S4 HPLC Chromatograms for folding reactions of μ-SIIIA. S14

S5 HPLC Chromatograms for folding reactions of α-ImI. S14

S6 Phylogenetic tree for the O1 superfamily, showing codon usage for Cysteine 1. . . . . S16

S7 Phylogenetic tree for the O1 superfamily, showing codon usage for Cysteine 2. . . . . S17

S8 Phylogenetic tree for the O1 superfamily, showing codon usage for Cysteine 3. . . . . S18

S9 Phylogenetic tree for the O1 superfamily, showing codon usage for Cysteine 4. . . . . S19

S10 Phylogenetic tree for the O1 superfamily, showing codon usage for Cysteine 5. . . . . S20

S11 Phylogenetic tree for the O1 superfamily, showing codon usage for Cysteine 6. . . . . S21

List of Tables

S1 Codon usage counts for site 10 in multiple sequence alignment of published ω-conotoxin genes. S7

S2 $\hat{N}_e(j)$ at site 10 of a multiple sequence alignment of published ω-conotoxins for amino acids that can be represented by two possible codons and their relative weights. S8

S3 Determination of the effective number of codons per amino acid for amino acids with 4 possible codons (Λ_4) at site 10 in a multiple sequence alignment of ω-conotoxins. S9

S4 Determination of the effective number of codons per amino acid for amino acids with 6 possible codons (Λ_6) at site 10 in a multiple sequence alignment of ω-conotoxins. S10

S1 Determination of the Effective Number of Codons per Amino Acid by Site

S1.1 Rationale

In order to consider codon-level conservation within a family of homologous sequences (especially from a single genus, such as *Conus*), it is important to be able to account for the homology that exists among this group of sequences. However, accounting for this homology is somewhat non-trivial, as the ancestral sequence is usually unknown, and in some cases cannot be readily determined.

While previous studies have not accounted for homology [1], and consequently resulted in extreme site-specific codon bias ($P < 1 \cdot 10^{-4}$), we decided to reassess the level of codon bias in a manner that accounts for homology between the sequences considered. However, because we do not necessarily know the origins or extent of that homology, we do not want to directly include any statements of what that homology may be in our analyses.

Herein we present a tool to assess codon bias site-by-site across a family of sequences, normalizing out the identity of any encoded amino acid, and without incorporating any assumptions about amino acids that are missing or underrepresented. However, in removing assumptions about missing amino acids, we cannot readily compile the redundancy classes (amino acids that can be encoded by the same number of codons, either 1, 2, 3, 4 or 6) into a single numeric output, and therefore must consider each redundancy class separately.

We have based this novel tool on the 'Effective Number of Codons,' originally introduced by Frank Wright [2]. In his work, the objective was to look at codon bias in a gene, in order to assess translational efficiency; other codon bias analyses have taken the same approach [3]. One concern is whether these methods can be used to analyze a relatively small sample set; of five prominent methods, the 'Effective Number of Codons' was among the least biased by short gene sequences [3], analogous to a relatively small set of sequences when considered site-by-site.

While our method seems intuitively similar to measuring a $\mathrm{D}_n/\mathrm{D}_s$ ratio because both measure the correlative capacity of amino acid to a codon at each site, the two differ in a few critical ways. $\mathrm{D}_n/\mathrm{D}_s$ is used to determine the evolutionary pressures on a gene, and what type of selection (purifying or adaptive) existed to result in the changes in that gene [4]. In contrast, our method does not have this power; rather,

it is a means to assess codon bias by site. Our method should serve as a bridge between measures of evolutionary pressures and conventional measures of codon bias; by allowing a site-specific measure of codon variability, it can provide evolutionary hints as to which portions of a peptide sequence are under different selective pressures.

S1.2 Overview of Adaptations to Effective Number of Codons

In the original treatment, the 'Effective Number of Codons' was determined by first finding the 'homozygosity' of each amino acid, which was then averaged within each redundancy class, and then the sum of the reciprocals is taken, weighted by the number of amino acids in each redundancy class. The result is a number between 20 and 61, which indicates the number of codons that are effectively used within that gene, accounting for the extent of bias for each of the 20 amino acids. If an amino acid is not present or underrepresented in a gene, then it is assumed that the codon bias for that amino acid would be the same as for the other amino acids within that redundancy class.

In our treatment, we sought to eliminate any assumptions about amino acids that were not present or underrepresented, as the sequence alignment for conotoxins contains numerous cysteine-only sites. Consequently, when considering each site, we instead used the effective number of codons *for each amino acid*, and then averaged these, weighting by the prevalence of that amino acid at that site (within that redundancy class). This results in a set of numbers for each redundancy class, at each site, allowing comparisons of codon bias between two sites using values that are intrinsically meaningful (for instance, for Site 4 in ω-conotoxins, amino acids that can be encoded by two codons are actually encoded by 1.059 codons, indicating marked codon bias at this site).

However, it is worth noting that comparisons between different redundancy classes cannot be readily made, as a consequence of our removing any assumptions about amino acids that are not present or underrepresented. Consequently, we cannot readily compare the codon bias at Site 4 in ω-conotoxins for amino acids that can be encoded by two codons (effectively, 1.059 codons) with the codon bias at the same site for amino acids that can be encoded by six codons (effectively, 2.145 codons), despite both showing notable codon bias and their representing the same site in the same family of sequences.

S1.3 Theoretical Buildup

For clarity, we use different indices to designate different 'levels' of information: i is for codon-level information; j is for amino acid level information; k is for redundancy class information. Although we only consider one at a time, in some cases more than one index is shown in order to clearly show the origins of each calculation.

To begin, each amino acid is placed into a "redundancy class," which designates the number of distinct, synonymous codons that can encode that amino acid. In the standard genetic code, two amino acids (M, W) can be encoded by one codon; nine amino acids (C, N, D, Q, E, H, K, F, Y) can be encoded by two codons; isoleucine can be encoded by three codons; five amino acids (A, G, P, T, V) can be encoded by four codons, and three amino acids (R, L, S) can be encoded by six codons. This method can be readily applied to any non-standard genetic code, as long as the amino acids encoded by each codon are known.

The number of occurrences of each codon are then counted, giving n_i. Thus, $n = \sum_i n_i$ is the total number of occurrences of the selected amino acid, and the probability of codon i is simply the number of occurrences of that codon divided by the total number of occurrences of that amino acid, $p_i = \frac{n_i}{n}$. So, by definition, $\sum_i p_i = 1$.

For each amino acid, we then determine the 'homozygosity' of amino acid j, $\hat{F}_j$, as in [2]:

$$\hat{F}_j = \frac{n \cdot \sum_{i=1}^{k} p_i^2 - 1}{n - 1} \tag{S1}$$

It is worth noting at this point that because $p_i \in [0, 1]$, $p_i^2 \leq p_i$, and thus $\sum_i p_i^2 \leq 1$, indicating that $0 \leq \hat{F}_j \leq 1$; however, if $\hat{F}_j = 0$, then it is disregarded, as this can only result from very few instances of amino acid j at that site ($n \leq 2$), indicating that amino acid is underrepresented and should be excluded from the analysis at this site.

The homozygosity of each amino acid ($\hat{F}_j$) is readily translated into the number of equally frequent codons that would show the same degree of homozygosity for that amino acid ($\hat{N}_e(j)$) by taking the reciprocal (as in [2]),

$$\hat{N}_e(j) = \frac{1}{\hat{F}_j}. \tag{S2}$$

Here is where we diverge from Wright's treatment (except that we were grouping sets of codons by site

across a family of genes, rather than in a gene). Since there is significant bias towards certain residues at certain sites in our alignment (for instance, cysteine residues at cysteine sites, or the site used in the sample calculation), we decided to use a weighted average of $\hat{N}_e(j)$ values, averaged within each redundancy class (however, the redundancy classes cannot be compared, in order to avoid assumptions about amino acids that are not present or underrepresented).

In order to determine a weighted average, we define a new coefficient, p_j, as the fraction of redundancy class k that encodes amino acid j (this is analogous to p_i, except that it uses amino acid level information, indicated by the j index), in terms of counts of the codons at that site. Using this, we can determine the number of equally frequent codons that would show the same level of homozygosity for that redundancy class, or the effective number of codons per amino acid at that site (within a redundancy class),

$$\Lambda_k = \sum_{j=1}^{l} (p_j \cdot \hat{N}_e(j)) \tag{S3}$$

where l is the number of non-excluded members of the redundancy class (exclusion as described for Eq. S1).

Λ_k can be interpreted as the effective number of (or, number of equally frequent) codons that are actually used at that site, for that redundancy class. So for redundancy class k, a value of 1 would indicate that only one codon was used for each amino acid, whereas a value of k would indicate that every available codon was used indiscriminately for each amino acid.

S1.4 Sample Calculation

For the sample calculation, we use the data from site 10 (counting from the first cysteine, as in Figure 2 in the main text, and Figures S1 and S2) of the published ω-conotoxin genes in a multiple sequence alignment.

First, the codons for each amino acid are counted, and placed into bins based on the redundancy classes. We will deal with each redundancy class separately; we will not discuss the non-redundant class (M and W) because these can only be encoded by one codon, and therefore $\Lambda_1 = 1$.

We will first address redundancy class 2, whose values are shown in Table S1. Not all amino acids are shown, but if there is an occurrence of that amino acid, all possible codons for that amino acid are shown.

Amino Acid	Codon	Count
N	AAT	7
	AAC	0
D	GAT	2
	GAC	0
Q	CAA	9
	CAG	1
H	CAT	11
	CAC	0
K	AAA	0
	AAG	13
F	TTT	8
	TTC	0
Y	TAT	22
	TAC	2

Table S1. Codon usage counts for site 10 in multiple sequence alignment of published ω-conotoxin genes.

The next step is to determine the homozygosity for each amino acid. Using the data for glutamine because there are instances of both codons, $p_{CAA} = \frac{9}{10}$ and $p_{CAG} = \frac{1}{10}$, so,

$$\hat{F}_Q = \frac{10 \cdot \sum_{i=1}^{2} p_i^2 - 1}{10 - 1} = \frac{10((\frac{9}{10})^2 + (\frac{1}{10})^2) - 1}{10 - 1} = \frac{10(\frac{82}{100}) - 1}{10 - 1} = \frac{7.2}{9} = 0.8$$

For any amino acid that has occurrances of only one of the codons, the homozygosity is one, since one of the p_i values is one, and the other(s) are 0. So, doing the calculation for tyrosine will tell us the homozygosity values for all of the 2-fold redundant amino acids at site 10 in ω-conotoxins:

$$\hat{F}_Y = \frac{24 \cdot \sum_{i=1}^{2} p_i^2 - 1}{24 - 1} = \frac{24((\frac{22}{24})^2 + (\frac{2}{24})^2) - 1}{24 - 1} = \frac{24(\frac{122}{144}) - 1}{24 - 1} = 0.84058.$$

Now, adding the homozygosity, the number of equally frequent codons (the reciprocal of $\hat{F}_j$), and the fraction of each redundancy class that is the given amino acid (p_j) to Table S1 results in Table S2.

And, taking the product of the last two columns, and summing the results, we get the number of equally frequent codons per amino acid at site 10 of the ω-conotoxins, for the amino acids that can be represented by two (and only two) codons,

Amino Acid	Codon	Count	$\hat{F}_j$	$\hat{N}_e(j)$	p_j
N	AAT	7	1	1	$\frac{7}{75}$
	AAC	0			
D	GAT	2	1	1	$\frac{2}{75}$
	GAC	0			
Q	CAA	9	0.8	1.25	$\frac{10}{75}$
	CAG	1			
H	CAT	11	1	1	$\frac{11}{75}$
	CAC	0			
K	AAA	0	1	1	$\frac{13}{75}$
	AAG	13			
F	TTT	8	1	1	$\frac{8}{75}$
	TTC	0			
Y	TAT	22	0.84058	1.1897	$\frac{24}{75}$
	TAC	2			

Table S2. **$\hat{N}_e(j)$ at site 10 of a multiple sequence alignment of published ω-conotoxins for amino acids that can be represented by two possible codons and their relative weights.**

$$\Lambda_2 = \sum_{j=1}^{l}(p_j \cdot \hat{N}_e(j)) = 1(\frac{7}{75} + \frac{2}{75} + \frac{11}{75} + \frac{13}{75} + \frac{8}{75}) + 1.25(\frac{10}{75}) + 1.1897(\frac{24}{75}) = 1.094.$$

To do the same calculation for the other redundancy classes, we show only the resultant final table; the calculations at each step are exactly the same as those above, but the sums are longer because of the increased number of codons for each amino acid.

For the redundancy class that includes only isoleucine, only one codon was used. These calculations are not shown because they reduce to the trivial case.

Note that there is only one instance of threonine, so this data point is excluded from the analysis.

The data for amino acids that can be encoded by four possible codons is shown in Table S3.

The same can be done for the amino acids encoded by six possible codons, and is shown in Table S4.

S1.5 Data for Redundancy Classes 4 and 6

In order to compare the relative conservation of cysteine to the other amino acids in the intercysteine loops, only the 2-fold redundant codons were used for analysis in the main paper. However, every redundancy class was analyzed for each site, in order to assess the extent of codon conservation within each redundancy class.

For almost every site, isoleucine was not sufficiently prevalent to merit analysis of the redundancy

Redundnacy Class 4						
**Amino Acid**	**Codon**	**Count**	$\hat{F}_j$	$\hat{N}_e(j)$	p_j	Λ_4
A	GCT	25	0.92308	1.0833	$\frac{26}{120}$	1.1883
	GCC	0				
	GCA	1				
	GCG	0				
G	GGT	55	1	1	$\frac{55}{120}$	
	GGC	0				
	GGA	0				
	GGG	0				
P	CCT	8	0.49474	2.0213	$\frac{20}{120}$	
	CCC	0				
	CCA	0				
	CCG	12				
T	ACT	1	N/A	N/A	Not Incl.	
	ACC	0				
	ACA	0				
	ACG	0				
V	GTT	19	1	1	$\frac{19}{120}$	
	GTC	0				
	GTA	0				
	GTG	0				

Table S3. Determination of the effective number of codons per amino acid for amino acids with 4 possible codons (Λ_4) at site 10 in a multiple sequence alignment of ω-conotoxins.

Redundnacy Class 6						
**Amino Acid**	**Codon**	**Count**	$\hat{F}_j$	$\hat{N}_e(j)$	p_j	Λ_6
R	CGT	28	0.51800	1.9305	$\frac{72}{130}$	1.9601
	CGC	0				
	CGA	0				
	CGG	0				
	AGA	0				
	AGG	0				
L	TTA	3	0.33846	2.9545	$\frac{26}{130}$	
	TTG	5				
	CTT	4				
	CTC	14				
	CTA	0				
	CTG	0				
S	TCT	2	0.82056	1.2187	$\frac{32}{130}$	
	TCC	0				
	TCA	1				
	TCG	0				
	AGT	29				
	AGC	0				

Table S4. Determination of the effective number of codons per amino acid for amino acids with 6 possible codons (Λ_6) at site 10 in a multiple sequence alignment of ω-conotoxins.

class with three possible codons. While these calculations were performed, they are excluded from this presentation because we believe them to be artificially skewed by small sample size effects.

In Figures S1 and S2, the vertical axes are scaled to the maximum possible value, which would represent indiscriminate usage of every possible codon for that amino acid. In most cases, the actual values are markedly lower than the maximum possible value, indicating that there is a global codon bias across all redundancy classes at almost every site. The bars are also color-coded by the number of sequences included in the analysis at that site, to indicate the risk of small sample size effects at the specified site for the specified redundancy class. Red or blue indicates that at least 30 sequences were analyzed; grey indicates that between 15 and 30 sequences were analyzed; white indicates that fewer than 15 sequences were analyzed.

S2 HPLC Data From Microsome-Assisted Folding

The differences between the ATP-depleted microsome folding reactions and the control (non-ATP-depleted) microsome folding reactions was subtle. However, the differences based on whether the folding reactions

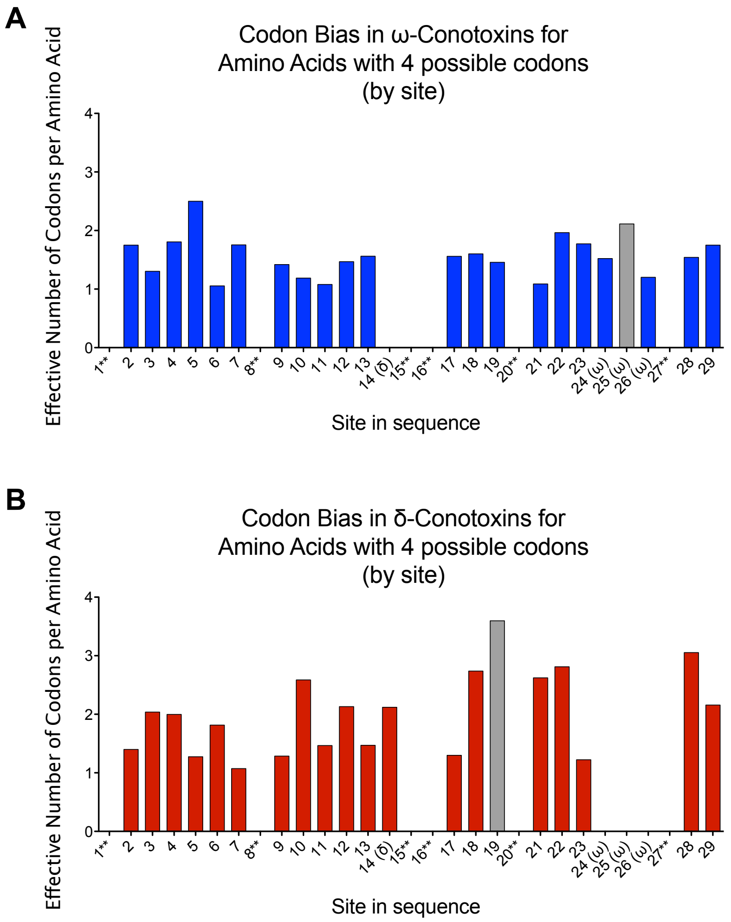

Figure S1. Codon bias in the ω- and δ-conotoxins, showing the bias for amino acids that can be represented by 4 possible codons. (A) shows the data for the ω-conotoxins, and **(B)** shows the data for the δ-conotoxins. Because the length of the intercysteine loops differ between these two classes of conotoxins, residues that are specific to each class are marked with the class to which they are specific, as in Figures 2 and 3 of the main paper. ** indicates cysteine sites, which are blank because there are two possible codons for cysteine. A value of 1 indicates that only one of the four possible codons is used for each residue, and a value of 4 would indicate that each of the four possible codons for each amino acid are used indiscriminately. Bars are color-coded to the size of the sample set being analyzed (the number of sequences with 4-fold redundant codons at that site): blue/red indicates at least 30 sequences and grey indicates between 15 and 30 sequences.

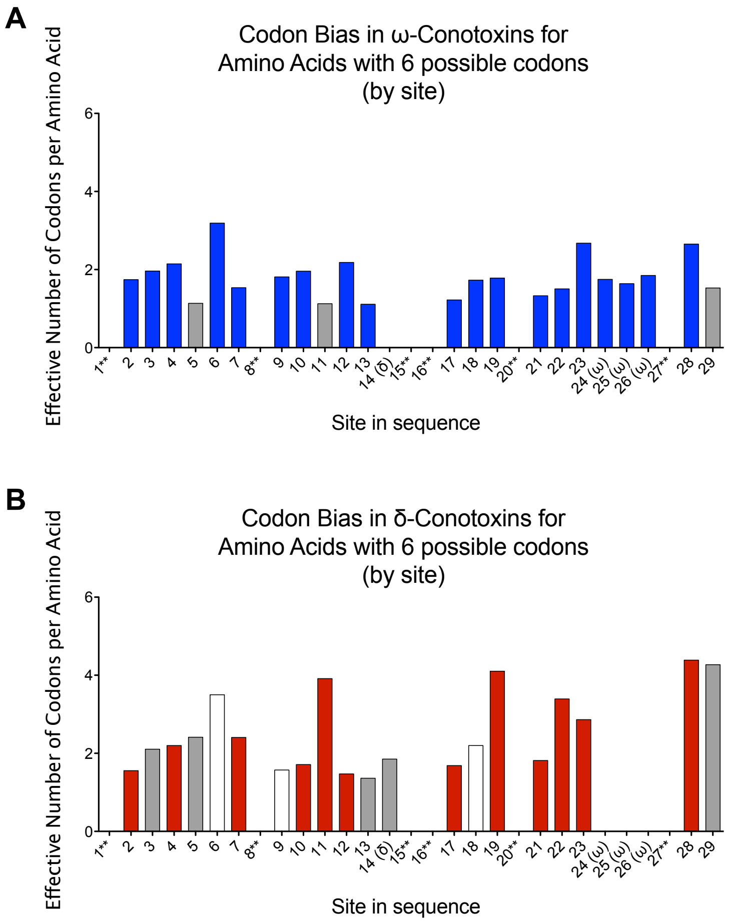

Figure S2. Codon bias in the ω- and δ-conotoxins, showing the bias for amino acids that can be represented by 6 possible codons. (A) shows the data for the ω-conotoxins, and **(B)** shows the data for the δ-conotoxins. Because the length of the intercysteine loops differ between these two classes of conotoxins, residues that are specific to each class are marked with the class to which they are specific, as in Figures 2 and 3 of the main paper. ** indicates cysteine sites, which are blank because there are two possible codons for cysteine. A value of 1 indicates that only one of the six possible codons is used for each residue, and a value of 6 would indicate that each of the six possible codons for each amino acid are used indiscriminately. Bars are color-coded to the size of the sample set being analyzed (the number of sequences with 6-fold redundant codons at that site): blue/red indicates at least 30 sequences, grey indicates between 15 and 30 sequences, and white indicates fewer than 15 sequences.

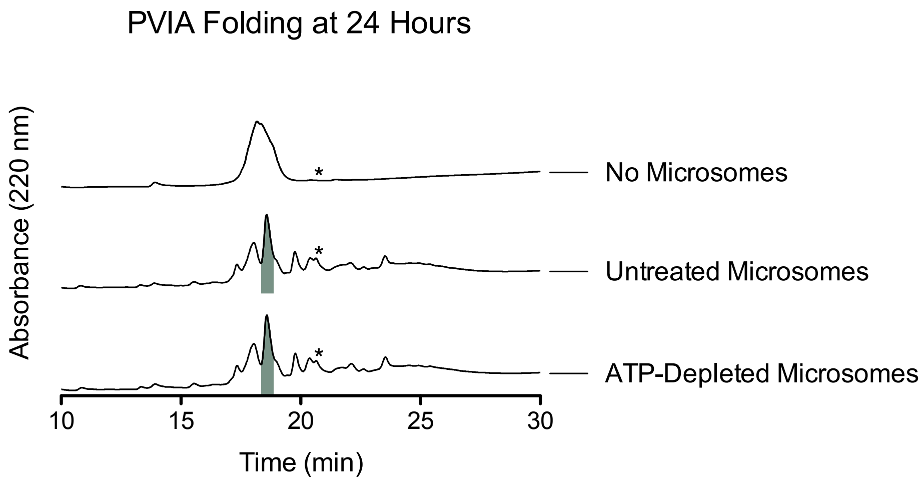

Figure S3. HPLC Chromatograms for folding reactions of δ-PVIA. All folding reactions come from a single run, in order to best enable comparison between conditions. The shown HPLC Chromatograms were collected on a Waters e2695 Separations Module, and were monitored at 220 nm. Samples were run on a diphenyl column (Vydac, 219TP54). Olive green integration is shown for the 'linear' or reduced form of the peptide, and * indicates the 'native' or folded form. The linear form of the No Microsomes condition is not integrated because the increased width of this peak exhibited shoulders that were not reproducible.

had microsomes at all were often quite pronounced. These differences are quantified in Table 1 in the main text. In order to more clearly demonstrate the subtle differences between the various conditions, exemplary HPLC chromatograms for each of the folding reactions are shown in Figures S3, S4 and S5. The timepoints shown in Figures S3, S4 and S5 are the same timepoints that are quantified in Table 1 of the main text. We note that these timepoints are *not* the terminal timepoints, but rather represent the most significant differences between the ATP-depleted and untreated microsome-assisted folding reactions.

S3 Phylogenetic Trees for O1 Superfamily

The following phylogenetic trees show the toxin diversification for the O1 superfamily, using all published sequences (see Section S4). As mentioned in the main text, it is evident from these trees that there is no counter-selection to revert to the canonical cysteine residue at that site.

Each tree shows the codon usage for a single cysteine. The tree for cysteine 1 is shown in Figure S6; cysteine 2 is shown in Figure S7; cysteine 3 is shown in Figure S8; cysteine 4 is shown in Figure S9;

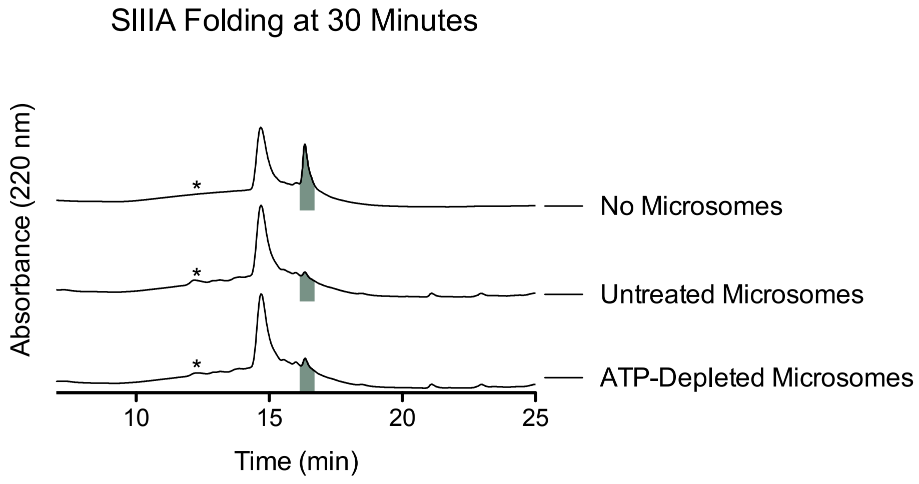

Figure S4. HPLC Chromatograms for folding reactions of μ-SIIIA. All folding reactions come from a single run, in order to best enable comparison between conditions. The shown HPLC Chromatograms were collected on a Waters e2695 Separations Module, and were monitored at 220 nm. Samples were run on a diphenyl column (Vydac, 219TP54). Olive green integration is shown for the 'linear' or reduced form of the peptide, and * indicates the 'native' or folded form.

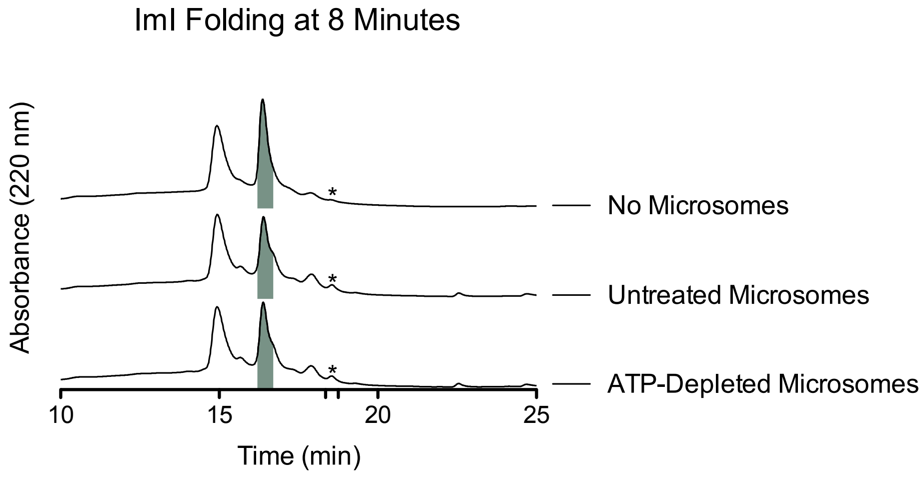

Figure S5. HPLC Chromatograms for folding reactions of α-ImI. All folding reactions come from a single run, in order to best enable comparison between conditions. The shown HPLC Chromatograms were collected on a Waters e2695 Separations Module, and were monitored at 220 nm. Samples were run on a diphenyl column (Vydac, 219TP54). Olive green integration is shown for the 'linear' or reduced form of the peptide, and * indicates the 'native' or folded form.

cysteine 5 is shown in Figure S10; cysteine 6 is shown in Figure S11. Each of these trees differs from the others only in which lines are hollow/shaded, indicating which cysteine codon is used at that site for the indicated conotoxin, allowing facile comparison of the evolutionary relationships of the codon usage patterns for each cysteine.

S4 Sequences Used for Analysis

S4.1 ω-Conotoxin Sequences

We used published sequences of ω-conotoxins for these analyses. The GenBank accession numbers for these sequences are (349 total sequences):

DQ141146.1, **DQ141149.4**, **AF090041.1**, **AF090042.1**, **AF090043.1**, **AF090044.1**, **AF090045.1**, **AF090046.1**, **AF090047.1**, **AF090048.1**, **AF090050.1**, **AF090051.1**, **AF090052.1**, **AF090054.1**, **AF090073.1**, **DQ644547.1**, **EF108269.1**, **EF108272.1**, **AF090055.1**, **AF090056.1**, **AF090057.1**, **AF090058.1**, **AF090059.1**, **AF090060.1**, **AF090061.1**, **AF090063.1**, **AF090064.1**, **AF090065.1**, **AF090066.1**, **AF090067.1**, **AF090068.1**, **AF090069.1**, **AF090071.1**, **AF090072.1**, **DQ644546.1**, **EU423370.1**, **EU423371.1**, **EU423372.1**, **EU423373.1**, **EU423374.1**, **EU423375.1**, **EU423376.1**, **EU423377.1**, **EU423378.1**, **EU423379.1**, **EU423380.1**, **EU423381.1**, **EU423382.1**, **EU423383.1**, **AF215040.1**, **AF215041.1**, **AF215042.1**, **DJ379450.1**, **AF089971.1**, **AF089972.1**, **AF089973.1**, **AF089974.1**, **AF089975.1**, **AF089976.1**, **AF089977.1**, **AF089978.1**, **AF089979.1**, **AF089980.1**, **AF089981.1**, **AF089982.1**, **AF089983.1**, **AF089984.1**, **AF089985.1**, **AF089986.1**, **AF089987.1**, **AF089988.1**, **AF089989.1**, **AF089990.1**, **AF089991.1**, **AF089992.1**, **AF089993.1**, **AF089995.1**, **AF089996.1**, **AF089997.1**, **AF089998.1**, **AF089999.1**, **AF090000.1**, **AF090001.1**, **AF090002.1**, **AF090003.1**, **AF090004.1**, **AF090005.1**, **AF090006.1**, **AF090007.1**, **AF090008.1**, **AF090009.1**, **AF090010.1**, **AF090011.1**, **AF090012.1**, **AF090013.1**, **AF090014.1**, **AF090015.1**, **AF090016.1**, **AF090017.1**, **AF090018.1**, **AF090019.1**, **AF090020.1**, **AF090021.1**, **AF090022.1**, **AF090023.1**, **AF090024.1**, **AF090025.1**, **AF090026.1**, **AF090027.1**, **AF090028.1**, **AF090029.1**, **AF090030.1**, **AF090031.1**, **AF090032.1**, **AF090033.1**, **AF090035.1**, **AF090036.1**, **AF090037.1**, **AF090038.1**, **AF090039.1**, **AF090053.1**, **AF090049.1**, **AF174268.1**, **AF174269.1**, **AF174271.1**, **AF174272.1**, **AF174273.1**, **AF174274.1**, **AF174275.1**, **AF174276.1**, **AF174277.1**, **AF174278.1**, **AF174279.1**,

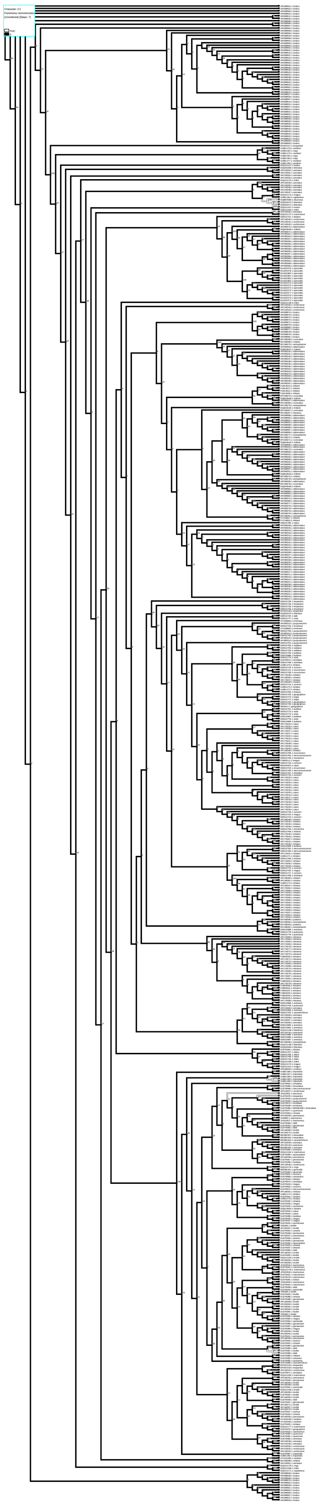

Figure S6. Phylogenetic tree for the O1 superfamily, showing codon usage for Cysteine 1. Hollow bars indicate TGC; shaded bars indicate TGT.

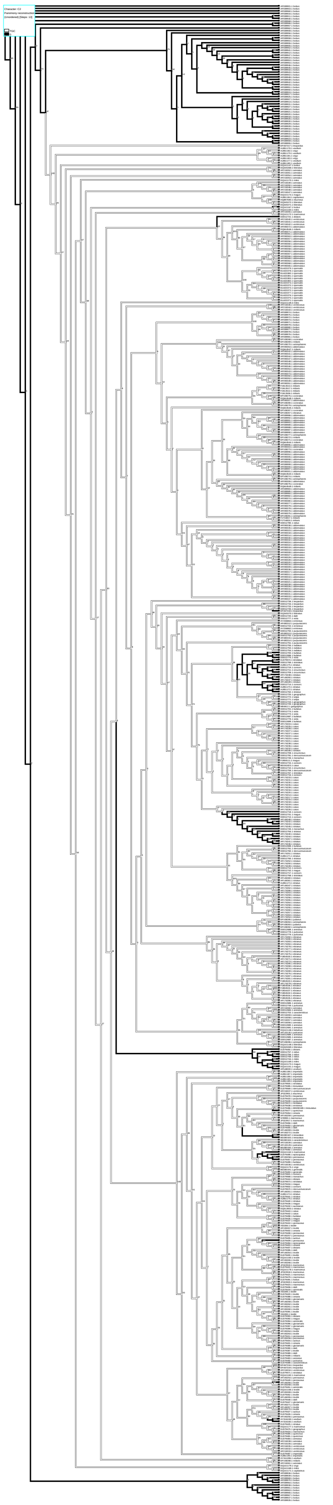

Figure S7. Phylogenetic tree for the O1 superfamily, showing codon usage for Cysteine 2. Hollow bars indicate TGC; shaded bars indicate TGT.

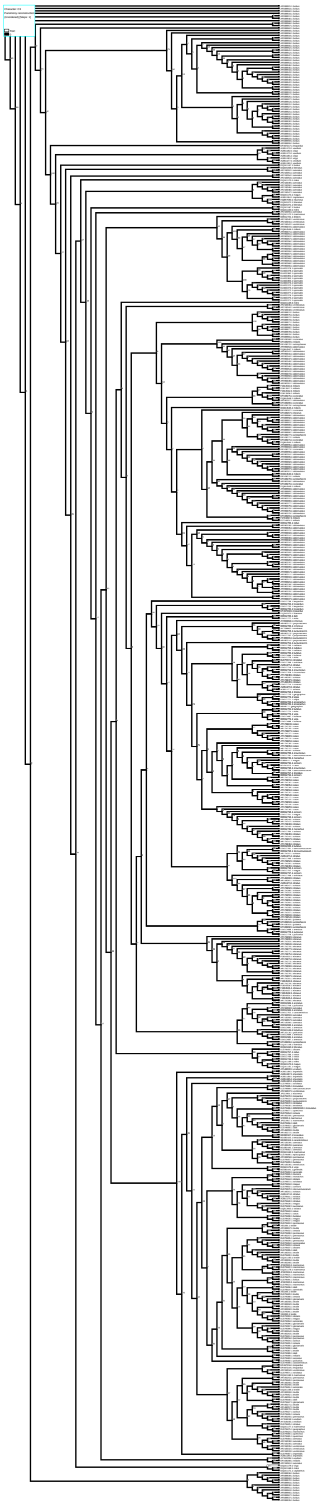

Figure S8. Phylogenetic tree for the O1 superfamily, showing codon usage for Cysteine 3. Hollow bars indicate TGC; shaded bars indicate TGT.

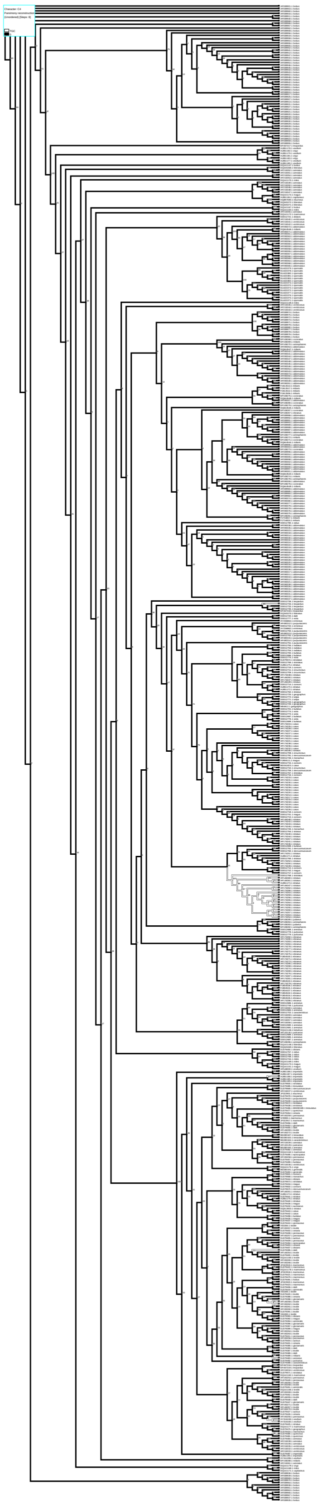

Figure S9. Phylogenetic tree for the O1 superfamily, showing codon usage for Cysteine 4. Hollow bars indicate TGC; shaded bars indicate TGT.

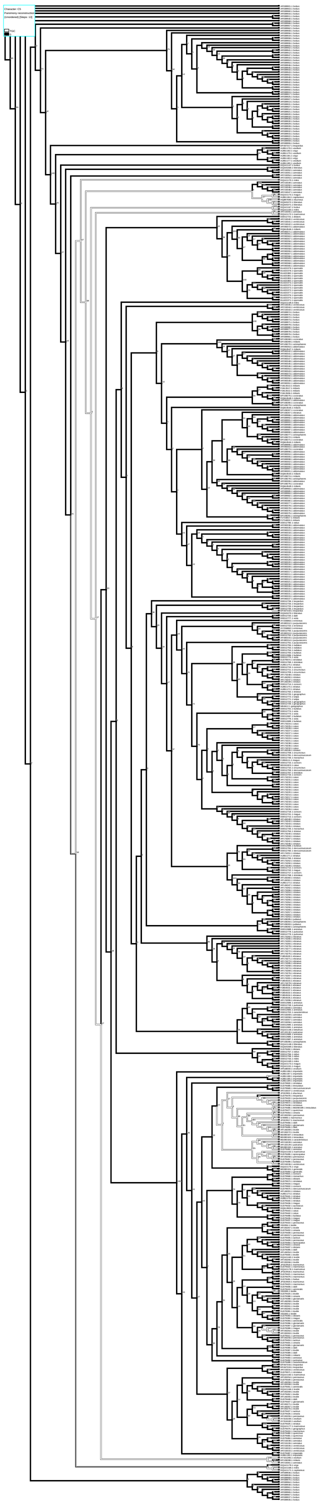

Figure S10. Phylogenetic tree for the O1 superfamily, showing codon usage for Cysteine 5. Hollow bars indicate TGC; shaded bars indicate TGT.

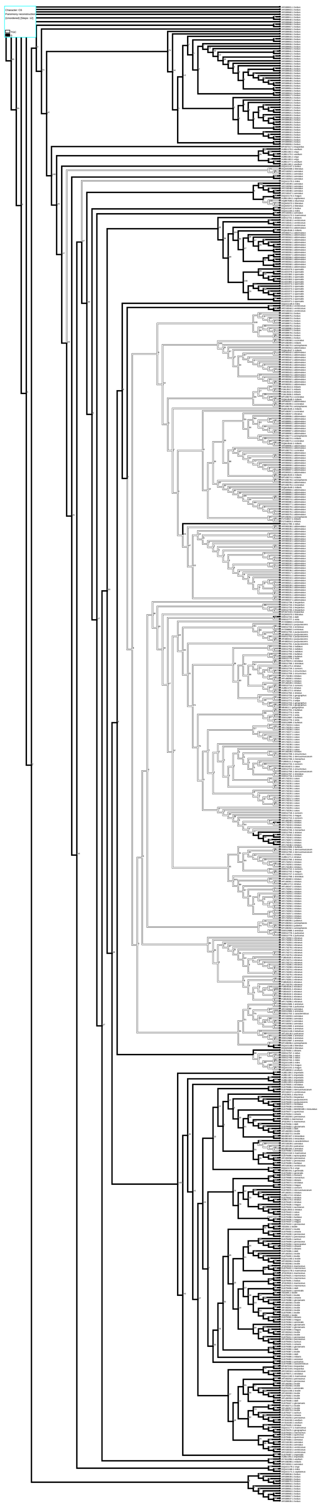

Figure S11. Phylogenetic tree for the O1 superfamily, showing codon usage for Cysteine 6. Hollow bars indicate TGC; shaded bars indicate TGT.

AF174280.1, **AF174281.1**, **AF174282.1**, **AF174283.1**, **AF174284.1**, **AF174285.1**, **AF174286.1**, **AF174287.1**, **FJ613506.1**, **FJ613512.1**, **FJ613513.1**, **FJ613517.1**, **FJ716817.1**, **FJ716824.1**, **FJ804530.1**, **FJ804531.1**, **FJ804532.1**, **FJ804533.1**, **FJ804534.1**, **FJ804535.1**, **FJ804536.1**, **FJ834437.1**, **AF090074.1**, **AF090075.1**, **AF090076.1**, **AF090077.1**, **AF090078.1**, **AF090079.1**, **AF090080.1**, **DQ644543.1**, **DQ644544.1**, **DQ644545.1**, **DQ644548.1**, **DQ644549.1**, **EF108267.1**, **EF108268.1**, **EF108270.1**, **EF108271.1**, **EF108273.1**, **EF108274.1**, **EF108275.1**, **EF108276.1**, **EF108277.1**, **EF108278.1**, **EF108279.1**, **EF108280.1**, **EF108281.1**, **EF108282.1**, **EF108283.1**, **EF108284.1**, **EF108285.1**, **EF108286.1**, **EF108287.1**, **AF132130.1**, **AF215060.1**, **AF215057.1**, **DD012749.1**, **DD012687.1**, **DD012688.1**, **DD012685.1**, **DD012686.1**, **DD012689.1**, **DD012778.1**, **DD012779.1**, **DD012780.1**, **DQ141148.1**, **DQ345369.1**, **AJ851170.1**, **AF146349.1**, **AJ851172.1**, **DD012762.1**, **DD012714.1**, **AF174214.1**, **AF174215.1**, **AF174216.1**, **AF174217.1**, **AF174218.1**, **AF174219.1**, **AF174220.1**, **AF174221.1**, **AF174222.1**, **AF174223.1**, **AF174224.1**, **AF174225.1**, **AF174226.1**, **AF174227.1**, **AF174228.1**, **AF174229.1**, **AF174230.1**, **AF174231.1**, **AF174232.1**, **AF174233.1**, **AF174235.1**, **AF174236.1**, **AF174237.1**, **AF174238.1**, **AF174239.1**, **AF174240.1**, **AF174241.1**, **AF174242.1**, **AF174243.1**, **AF174244.1**, **AF174245.1**, **AF174246.1**, **AF174247.1**, **AF174248.1**, **AJ851175.1**, **AF146350.1**, **DD012769.1**, **DD012696.1**, **DD012700.1**, **DD012775.1**, **DD012711.1**, **DD012719.1**, **DJ379574.1**, **DD012709.1**, **AF480312.1**, **DD012713.1**, **AF174267.1**, **DD012697.1**, **AF146348.1**, **AF146346.1**, **DD012710.1**, **DD012751.1**, **M84612.1**, **FJ959111.1**, **DD012715.1**, **DD012741.1**, **DD012745.1**, **DD012746.1**, **DD012764.1**, **DD012767.1**, **DD012726.1**, **DD012699.1**, **DD012701.1**, **DD012776.1**, **DD012708.1**, **DD012755.1**, **DD012759.1**, **DD012773.1**, **DD012716.1**, **DD012718.1**, **DD012724.1**, **DD012725.1**, **DD012781.1**, **BD241823.1**, **DD012772.1**, **DD012771.1**, **DD012774.1**, **DD012750.1**, **DD012722.1**, **DD012752.1**, **AF480313.1**, **AF480314.1**, **AF480315.1**, **AY236862.1**, **AY236863.1**, **AF174249.1**, **AF174250.1**, **AF174251.1**, **AF174252.1**, **DD012760.1**, **DD012720.1**, **DD012698.1**, **AJ851171.1**, **DD012712.1**, **DD012742.1**, **DD012777.1**, **DD012736.1**, **DD012717.1**, **DD012765.1**, **DD012761.1**, **EF467318.1**, **DD012733.1**, **DD012734.1**, **DD012735.1**, **DQ345370.1**, **DD012753.1**, **DD012754.1**, **AF174253.1**, **AF174254.1**, **AF174255.1**, **AF174256.1**, **AF174257.1**, **AF174258.1**, **AF174259.1**, **AF174260.1**, **AF174262.1**, **AF174263.1**, **AJ851173.1**, **DD012768.1**, **AF146347.1**, **AF174264.1**, **AF146360.1**, **AF146361.1**, **AF174265.1**, **AF174266.1**, **AF215043.1**, **AF215044.1**, **AF215045.1**, **AF215056.1**, **AF215058.1**, **DD012690.1**, **DD012691.1**, **DD012702.1**, **DD012684.1**, **AF215059.1**, **DD012721.1**, **DD012756.1**, **DD012757.1**,

DD012758.1, **AF548000.1**, **DQ141151.1**, **DQ141169.1**, **DQ141173.1**, and **DD012744.1**.

S4.2 δ-Conotoxin Sequences

We used published sequences of δ-conotoxins for these analyses. The GenBank accession numbers for these sequences are (262 total sequences):

AF089901.1, **AF089902.1**, **AF089903.1**, **AF089904.1**, **AF089905.1**, **AF089906.1**, **AF089907.1**, **AF089908.1**, **AF089910.1**, **AF089911.1**, **AF089912.1**, **AF089913.1**, **AF089914.1**, **AF089915.1**, **AF089916.1**, **AF089917.1**, **AF089918.1**, **AF089919.1**, **AF089920.1**, **AF089921.1**, **AF089922.1**, **AF089923.1**, **AF089924.1**, **AF089925.1**, **AF089926.1**, **AF089927.1**, **AF089928.1**, **AF089929.1**, **AF089930.1**, **AF089931.1**, **AF089932.1**, **AF089933.1**, **AF089934.1**, **AF089935.1**, **AF089936.1**, **AF089937.1**, **AF089938.1**, **AF089939.1**, **AF089940.1**, **AF089941.1**, **AF089942.1**, **AF089943.1**, **AF089944.1**, **AF089945.1**, **AF089946.1**, **AF089947.1**, **AF089948.1**, **AF089949.1**, **AF089950.1**, **AF089951.1**, **AF089952.1**, **AF089953.1**, **AF089954.1**, **AF089955.1**, **AF089956.1**, **AF089957.1**, **AF089958.1**, **AF089959.1**, **AF089960.1**, **AF089961.1**, **AF089962.1**, **AF089963.1**, **AF089964.1**, **AF089965.1**, **AF089966.1**, **AF089967.1**, **AF089968.1**, **AF089969.1**, **AF089970.1**, **AF215054.1**, **EF467317.1**, **AJ851177.1**, **AJ851178.1**, **AJ851179.1**, **AJ851180.1**, **AJ851181.1**, **AJ851182.1**, **AJ851183.1**, **DQ141147.1**, **DQ345368.1**, **DQ141179.1**, **AF215051.1**, **AF215052.1**, **AF215046.1**, **AF215047.1**, **AF215048.1**, **AF215049.1**, **AF215050.1**, **AF215053.1**, **DQ141150.1**, **DQ141171.1**, **DQ141168.1**, **DQ141167.1**, **AJ851184.1**, **AF215055.1**, **DQ141174.1**, **DQ345371.1**, **DQ345372.1**, **HQ897690.1**, **DQ141170.1**, **AY151285.1**, **DQ141172.1**, **EF108288.1**, **AF215061.1**, **AF193254.1**, **AF146357.1**, **AF193255.1**, **AF193268.1**, **AF193269.1**, **DQ141166.1**, **DQ141177.1**, **AY316159.1**, **AY316160.1**, **AF193270.1**, **AF193271.1**, **DJ379427.1**, **DJ379425.1**, **DJ379448.1**, **DJ379475.1**, **DJ379447.1**, **DJ379424.1**, **DJ379426.1**, **DJ379452.1**, **AF146355.1**, **AF146356.1**, **DJ379449.1**, **DJ379451.1**, **AF146351.1**, **AF146354.1**, **AF193259.1**, **AF193272.1**, **AF146359.1**, **AF193260.1**, **AF193261.1**, **AF193263.1**, **AF193264.1**, **AF193265.1**, **AF193266.1**, **AF193267.1**, **AF215033.1**, **AF215034.1**, **AF215035.1**, **AF193256.1**, **AJ851190.1**, **AF193257.1**, **BD280191.1**, **AF215039.1**, **AJ851174.1**, **AJ851176.1**, **AJ851185.1**, **AJ851186.1**, **AJ851187.1**, **AJ851188.1**, **AJ851189.1**, **DJ379400.1**, **DJ379437.1**, **DJ379444.1**, **DJ379453.1**, **DJ379454.1**, **DJ379455.1**, **DJ379456.1**, **DQ141175.1**, **DJ379483.1**, **DJ379484.1**, **DJ379488.1**, **DJ379493.1**, **DJ379489.1**, **DJ379492.1**,

DJ379571.1, **DQ141160.1**, **DQ141178.1**, **DQ512803.1**, **JF322901.1**, **JF322917.1**, **JF322918.1**, **JF322919.1**, **JF322920.1**, **S78990.1**, **X53283.1**, **X53284.1**, **X53285.1**, **DJ379432.1**, **DJ379419.1**, **DJ379394.1**, **DJ379404.1**, **DJ379405.1**, **DJ379498.1**, **DJ379442.1**, **DJ379443.1**, **BD280184.1**, **DJ379502.1**, **DJ379501.1**, **DJ379395.1**, **DJ379406.1**, **DJ379393.1**, **DJ379407.1**, **DJ379438.1**, **DJ379494.1**, **AF215106.1**, **AF215038.1**, **EF467316.1**, **EF467319.1**, **DJ379396.1**, **DJ379397.1**, **DJ379385.1**, **DJ379487.1**, **DJ379420.1**, **DJ379421.1**, **DJ379422.1**, **DJ379423.1**, **DJ379478.1**, **DJ379491.1**, **DJ379434.1**, **DJ379392.1**, **DJ379399.1**, **DJ379435.1**, **DJ379436.1**, **DJ379568.1**, **DJ379479.1**, **DJ379401.1**, **DJ379402.1**, **DJ379403.1**, **DJ379388.1**, **DJ379439.1**, **DJ379408.1**, **DJ379409.1**, **DJ379410.1**, **DJ379411.1**, **DJ379433.1**, **DJ379570.1**, **DJ379441.1**, **DJ379440.1**, **DJ379500.1**, **DJ379572.1**, **DJ379573.1**, **DJ379485.1**, **DJ379391.1**, **DJ379486.1**, **BD280183.1**, **BD280187.1**, **AF215032.1**, **AF132129.1**, **DJ379482.1**, **BD280190.1**, **DQ141182.1**, **DJ379398.1**, **AF146358.1**, **AF193262.1**, **DJ379495.1**, **DJ379457.1**, **AF193258.1**, **DJ379480.1**, **DJ379481.1**, **DQ141162.1**, **AF215036.1**, **AF215037.1**, **DJ379499.1**, **DJ379569.1**, **DJ379477.1**, **DJ379389.1**, **DJ379390.1**, and **DJ379387.1**.

Supplemental References

1. Conticello SG, Gilad Y, Avidan N, Ben-Asher E, Levy Z, et al. (2001) Mechanisms for evolving hypervariability: the case of conopeptides. Mol Biol Evol 18: 120-131.

2. Wright F (1990) The 'effective number of codons' used in a gene. Gene 87: 23-29.

3. Comeron JM, Aguadé M (1998) An evaluation of measures of synonymous codon usage bias. J Mol Evol 47: 268-274.

4. Yang Z, Bielawski JP (2000) Statistical methods for detecting molecular adaptation. Trends Ecol Evol 15: 496-503.
